# Exploring the Vaccine Adjuvant Effect and Mechanism of *Epimedium* Using Network Pharmacology, Molecular Docking, and Molecular Dynamics Simulations

**DOI:** 10.3390/vaccines14050385

**Published:** 2026-04-26

**Authors:** Meng Tang, Anni Zhao, Yun Yang, Zhen Song, Sheng Wang, Xianghao Ye, Haozheng Luo, Liqun Zhao, Jiale Pan, Quanming Zou, Hongwu Sun, Hao Zeng

**Affiliations:** 1 Department of Microbiology and Biochemical Pharmacy, College of Pharmacy, Third Military Medical University, Chongqing 400038, China; tm200302@tmmu.edu.cn (M.T.); anne1224@tmmu.edu.cn (A.Z.); yy9008@hotmail.com (Y.Y.); songzhen@tmmu.edu.cn (Z.S.); wangs@tmmu.edu.cn (S.W.); yjsyxh@tmmu.edu.cn (X.Y.); luohz@tmmu.edu.cn (H.L.); zhaoliqun@tmmu.edu.cn (L.Z.); panjl@tmmu.edu.cn (J.P.); qmzou2007@163.com (Q.Z.); 2 State Key Laboratory of Trauma, Burn and Combined Injury, Third Military Medical University, Chongqing 400038, China

**Keywords:** *Epimedium*, vaccine adjuvant, network pharmacology, molecular docking, molecular dynamics simulations

## Abstract

**Background:** *Epimedium* is a natural herb with immunomodulatory potential, but its vaccine adjuvant properties remain poorly understood. **Objective:** The aim of this study was to elucidate the adjuvant effects of *Epimedium* and the underlying molecular mechanisms. Methods: Network pharmacology was used to identify bioactive compounds and targets of *Epimedium* from the TCMSP database, and immunomodulation-related targets from GeneCards and OMIM. PPI networks, KEGG/GO enrichment, molecular docking, and molecular dynamics (MD) simulations were performed. In vivo, female BALB/c mice were immunized with the *Staphylococcus aureus* (*S. aureus*) vaccine subunit HI antigen, either alone or with low- or high-dose icariin (ICA). Serum antibody responses (IgG, IgG1, IgG2a, IgG2b) were measured by ELISA. Survival against lethal *S. aureus* USA300 challenge was monitored. **Results:** Network pharmacology predicted 488 targets and 13 pathways. Core targets included IL6, TP53, EGFR, CTNNB1, HIF1A, HSP90AA1, JUN, MTOR, SRC, and AKT1. KEGG/GO analysis indicated involvement of T cell receptor and NOD-like receptor signaling pathways in inflammatory responses. Molecular docking and MD simulations confirmed stable ligand-target binding. Experimental validation showed that ICA significantly enhanced HI-specific antibody responses and induced a Th2-biased humoral immune response (IgG1/IgG2a ratio > 1), which is particularly relevant for vaccines targeting extracellular pathogens such as *S. aureus*. ICA also improved survival after lethal bacterial challenge. **Conclusions:** This study identifies potential bioactive compounds, core targets, and key pathways of *Epimedium* as a vaccine adjuvant. Experimentally, ICA, as a representative component, enhanced HI-specific antibody responses and conferred protection against lethal *S. aureus* challenge. Together, these findings offer a computational–experimental basis that may guide further mechanistic investigation.

## 1. Introduction

Vaccine adjuvants are essential compounds that enhance the immune response to vaccines by increasing the immunogenicity of antigens and promoting a durable, balanced humoral and cellular immune response [1,2]. Currently, the global vaccine adjuvant market is experiencing steady growth, driven by the rapid development of new vaccine technologies (e.g., mRNA vaccines, recombinant subunit vaccines) [3]. This growth is largely attributed to the increasing demand for enhanced vaccine efficacy, the accelerated development of vaccines for emerging infectious diseases, and the expanding immunotherapy field. However, the market is still primarily dominated by a limited range of commercialized adjuvants, such as aluminum salts, MF59, and the AS series. These adjuvants are characterized by a relatively narrow product variety, high patent barriers, long research and development timelines, and stringent regulatory approval processes [4].

Although traditional adjuvants, such as aluminum salts and oil emulsions, are widely used and hold significant market share, their immunological limitations are becoming increasingly evident. For example, aluminum adjuvants strongly induce humoral immunity but are often ineffective at eliciting Th1-type cellular immune responses. They may also cause local inflammation, allergic reactions, or even potential neurotoxicity [5]. However, for extracellular bacterial pathogens, such as *Staphylococcus aureus* (*S. aureus*), a Th2-biased antibody response may be sufficient or even advantageous, as opsonophagocytic killing is the primary protective mechanism [6]. Therefore, the ideal adjuvant should be selected based on the target pathogen and the desired immune outcome. There remains a clear need for adjuvants that can induce tailored immune responses—whether Th1, Th2, or balanced—while maintaining high efficacy, low toxicity, and controllable costs [7].

Given these circumstances, the exploration of natural herb adjuvants represents a crucial direction in current research, development, and market breakthroughs [8]. Natural herb adjuvants are favored for their high biocompatibility, diverse mechanisms of action, and potential safety advantages [9]. These adjuvants contain abundant immunologically bioactive compounds (e.g., polysaccharides, saponins, flavonoids) that have demonstrated excellent immunomodulatory potential in basic research [10]. Natural herb adjuvants can synergistically activate innate and adaptive immunity through multi-target and multi-pathway interactions, promoting antigen presentation and lymphocyte activation [11]. Moreover, they hold promise in enhancing immunogenicity while potentially reducing toxicity risks, which could address the shortcomings of existing adjuvants in terms of cellular immunity induction, immune durability, and safety.

Epimedium is a natural herb that has been used in traditional medicine for centuries. Its chemical composition is complex, containing bioactive compounds such as flavonoids, lignans, alkaloids, and volatile oils. Among these, flavonoids—particularly icariin (ICA) and its derivatives—are recognized as the primary group of bioactive compounds [12,13]. These compounds do not exist in isolation, but rather as a family sharing a common structural core. Modern pharmacological studies have extensively revealed that the natural herb’s broad biological activities, including immunomodulatory effects, arise from this complex and synergistic group of bioactive ingredients [14,15]. These compounds act through mechanisms that converge on multiple targets and pathways, fully embodying the characteristic systemic regulation of natural herbs [16,17]. In recent years, with increasing research into natural immunomodulators, accumulating evidence has indicated that Epimedium possesses significant immunoenhancing properties. In vitro and in vivo studies have shown that its main bioactive component, ICA, enhances the production and activation of CD4+ and CD8+ T cells, improves macrophage phagocytic activity, and strengthens antigen presentation by upregulating MHC-I molecules and related genes (LMP2, LMP7, TAP1, TAP2) [15]. According to traditional use and existing literature, Epimedium has shown acceptable tolerability within certain dose ranges, making it a promising candidate for vaccine adjuvant development [18]. Based on these findings, Epimedium, as a natural herb with both immunoenhancing and biosafety profiles, warrants further systematic exploration for its potential value in vaccine development.

However, its potential as a vaccine adjuvant and the underlying molecular mechanisms have not been systematically elucidated. Network pharmacology, a systems biology approach, can reveal the complex interactions between natural herbs and biological systems from a systems-level perspective, making it a powerful tool for deciphering pharmacological mechanisms [19,20]. By overcoming the limitations of the traditional “single-target, single-drug” model, its core advantage lies in constructing multi-layered biological networks and integrating computational predictions to comprehensively explain the holistic mechanisms of multi-component, multi-targets, and multi-pathway synergistic actions, particularly of natural herbs [21]. For example, a recent study combined network pharmacology prediction with experimental validation, demonstrating that anemoside B4 (AB4) exerts its adjuvant effect by targeting core molecules such as STAT3 and TLR4, modulating signaling pathways, including TLR4/NF-κB, thereby promoting immune organ development and enhancing both humoral and cellular immune responses [9]. This provides a paradigm for developing natural herbs adjuvants and serves as a crucial methodological bridge connecting traditional medical experience with modern science, thereby advancing the development of precision medicine. Additionally, molecular docking and molecular dynamics (MD) simulations, as computational tools, can theoretically predict and validate compound-targets interactions (including modes and affinities), offering theoretical support for network pharmacology predictions [22,23].

The rationale for selecting ICA as the representative compound for in vivo validation is as follows. Although Epimedium contains multiple bioactive compounds, ICA is the most abundant flavonoid glycoside, the pharmacopoeia-specified quality control marker, and the most extensively studied immunomodulatory component of this natural herb [13,24]. Numerous studies have demonstrated its ability to enhance T-cell activation, macrophage phagocytosis, and antigen presentation [25]. Therefore, selecting ICA as the representative compound for in vivo validation establishes a link between computational predictions and biological activity. This approach allows us to test whether the adjuvant effects predicted by network pharmacology can be experimentally observed using the most characteristic active ingredient of Epimedium.

**Figure 1:**
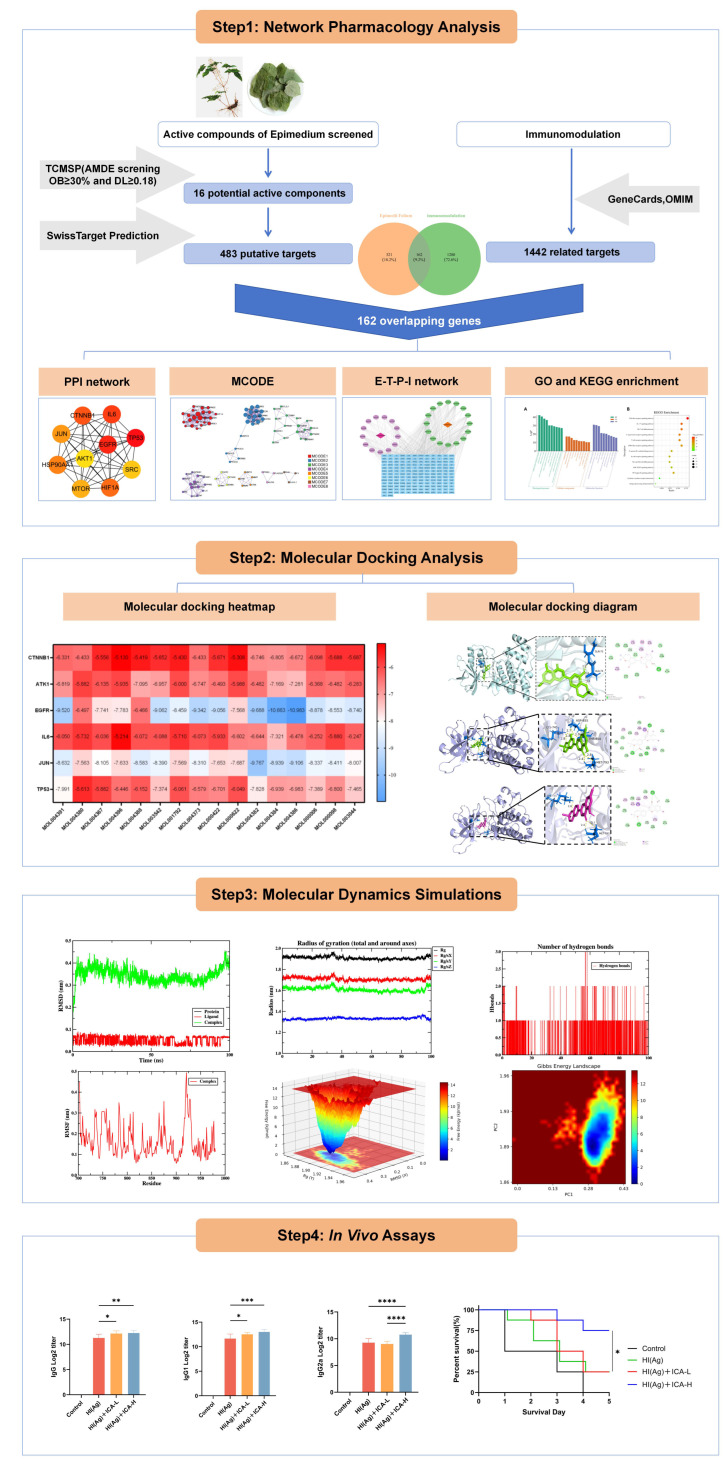
Flow chart showing the experimental design. The study consists of three phases: (1) Network pharmacology: screening of Epimedium bioactive compounds (TCMSP + SwissADME), target prediction (SwissTargetPrediction), immunomodulation-related genes collection (GeneCards/OMIM), PPI network construction (STRING), KEGG/GO enrichment, and “Epimedium-target-pathway-immunomodulation” network analysis. (2) Molecular docking and MD simulations: binding affinity evaluation and 100 ns dynamic simulation of ligand-target complexes. (3) In vivo validation: immunization of mice with HI antigen + ICA, measurement of HI-specific IgG subclasses by ELISA, and survival monitoring after lethal *S. aureus* USA300 challenge. * *p* < 0.05, ** *p* < 0.01, *** *p* < 0.001, **** *p* < 0.0001.

This study was designed to systematically explore the vaccine adjuvant potential of Epimedium and elucidate its possible mechanism of action as an immune adjuvant using network pharmacology, molecular docking, and molecular dynamics (MD) simulation (Figure 1). The key immunological features to be evaluated include HI-specific total IgG, IgG subclasses (IgG1, IgG2a, IgG2b), and the IgG1/IgG2a ratio, which serves as an indicator of Th1/Th2 balance. These parameters are critical for assessing adjuvant efficacy: total IgG reflects the overall enhancement of antigen immunogenicity; IgG1 indicates a Th2-biased humoral response that promotes antibody production, particularly relevant for protection against extracellular pathogens such as *S. aureus*; IgG2a reflects a Th1-biased response associated with cellular immunity; and the IgG1/IgG2a ratio informs about the functional polarization of the immune response. The HI antigen used in this study is a recombinant fusion protein comprising *S. aureus* α-hemolysin (Hla) and iron-regulated surface determinant B (IsdB), which elicits both B-cell and T-cell epitopes and serves as a well-established model antigen for adjuvant evaluation. The challenge strain, USA300, is a clinically relevant methicillin-resistant *S. aureus* isolate. Experimental validation that ICA, as a representative component of Epimedium, acts as an effective adjuvant for the *S. aureus* vaccine subunit HI antigen will be performed by measuring these antibody responses and challenging immunized mice with lethal *S. aureus* USA300 to assess protective immunity. This will provide functional evidence for antibody-mediated protection. Our research is expected to provide a theoretical foundation for the development of Epimedium as a novel vaccine adjuvant and offer new perspectives for in-depth research on natural herb adjuvants.

## 2. Materials and Methods

### 2.1. Acquisition of the Bioactive Compounds and Target Genes of Epimedium

Bioactive compounds from Epimedium were screened from the TCMSP database (https://tcmsp-e.com/, accessed on 2 January 2026) [26], a pharmacology resource detailing herb-compound-target interactions, was employed. Compounds were screened based on thresholds for drug-likeness (DL ≥ 0.18) and oral bioavailability (OB ≥ 30%) [27,28], with ADME properties further assessed using SwissADME (https://swissadme.ch/, accessed on 2 January 2026) [29,30].

These compounds were converted into standard SDF and SMILES formats using PubChem (https://pubchem.ncbi.nlm.nih.gov/, accessed on 2 January 2026) [31]. Subsequently, the SMILES formats of the compounds were used to explore the SwissTargetPrediction (https://swisstargetprediction.ch/, accessed on 2 January 2026) database for the identification of potential targets [32,33]. The targets collected through the above-mentioned methods were defined as the potential Epimedium targets.

### 2.2. Acquisition of the Potential Targets Genes of Immunomodulation

Furthermore, pertinent information regarding immunomodulation was retrieved from GeneCards and OMIM [34,35]. To improve the reliability of our predictions, only target proteins scoring within the top 10% of the GeneCards ranking were retained. After merging the results, duplicate entries were removed, and the remaining unique targets were designated as key immunomodulatory targets.

### 2.3. PPI Network Construction

To identify cross-targets, the potential Epimedium targets and immunomodulation-related targets were intersected using the Venny 2.1 online tool (https://bioinfogp.cnb.csic.es/tools/venny/, accessed on 2 January 2026). The overlapping targets were imported into the STRING database (https://cn.string-db.org/, accessed on 2 January 2026) [36,37], with the species restricted to Homo sapiens and an interaction score threshold set above 0.4. The derived protein–protein interaction (PPI) data were formatted and visualized as a network using Cytoscape 3.10.3 software [38].

### 2.4. Construction of the Epimedium-Target-Pathway-Immunomodulation Network

To map the systematic relationships between the bioactive compounds and targets, we built and visualized an integrated Epimedium-Target-Pathway-Immunomodulation network using Cytoscape 3.10.3.

### 2.5. KEGG Pathway and GO Functional Enrichment Analyses of Targets

The Metascape online tool (https://metascape.org/, accessed on 2 January 2026) was utilized to conduct KEGG pathway and GO functional enrichment analyses for the overlapping targets [39,40]. The analysis was performed with the species specified as “Homo sapiens”. The GO functional enrichment results, encompassing cellular components, molecular functions, and biological processes, were visualized using both bubble and bar charts generated by an integrated bioinformatics tool.

### 2.6. Molecular Docking

Potential immunomodulation targets prioritized via network pharmacology and enrichment analysis were subsequently investigated through molecular docking. The 3D structures of the target proteins were obtained from the Protein Data Bank (PDB, https://www.rcsb.org/, accessed on 2 January 2026) [41], whereas the structural data for the Epimedium ligand was acquired from PubChem. Molecular docking was conducted with the PrankWeb platform (https://prankweb.cz/, accessed on 2 January 2026), and the docking energy was calculated [42,43,44,45]. The optimal complex, defined by its minimal binding energy, was preserved as a PDB file for further analysis and visualization using PyMOL 3.0.3. Visualization result maps depicting molecular interactions were then exported.

### 2.7. MD Simulations

Following the docking outcomes, the complex was selected for MD simulation. The simulations were executed with the GROMACS 5.0 software [46]. employing the CHARMM36 force field [47] within a periodic boundary framework. The ligand topology was prepared and modeled with the CHARMM General Force Field [48], and the system charge was neutralized by introducing counterions. We employed the steepest descent algorithm for energy minimization to eliminate initial steric clashes.

The Particle Mesh Ewald (PME) method was applied to handle long-range electrostatic and van der Waals interactions. The system underwent a two-stage equilibration process: The system was first equilibrated under the NVT ensemble for 50,000 steps and then under the NPT ensemble for an additional 50,000 steps. Subsequently, a production MD run was carried out for 100 ns at 300 K, utilizing a 2.0 fs integration step, and coordinates saved every picosecond for subsequent analysis [49,50]. resulting xvg data files were processed and visualized with the qtGrace tool v0.2.6 (https://sourceforge.net/projects/qtgrace/, accessed on 2 January 2026).

### 2.8. Mice

Female BALB/c mice were purchased from Kangge Biotechnology Company (Chongqing, China) and housed in a specific-pathogen-free (SPF) facility. Mice used for in vitro experiments were 6–8 weeks of age, 18–20 g. Animal welfare and experimental procedures were carried out in accordance with the Guide for the Care and Use of Laboratory Animals and related ethical regulations of Army Medical University.

### 2.9. In Vivo Assays

#### 2.9.1. Preparation of ICA

ICA was dissolved in DMSO at a concentration of 5 × 10^4^ mg/L and stored at 4 °C.

#### 2.9.2. Immunization Grouping

All reagents used for immunization were filtered through 0.22 μm membranes prior to injection. A total of 48 female BALB/c mice were randomly divided into 6 groups (*n* = 8 per group). Mice were immunized with a prime-boost regimen: a primary immunization consisting of two doses, followed by a booster immunization one week later. For the primary immunization, mice were inoculated with the following formulations: Control (solvent alone), HI (Ag) (30 μg/mouse HI antigen alone), ICA-L (10 mg/kg ICA alone), ICA-H (20 mg/kg ICA alone), HI (Ag) + ICA-L (30 μg/mouse HI antigen + 10 mg/kg ICA), HI (Ag) + ICA-H (30 μg/mouse HI antigen + 20 mg/kg ICA). One week after the booster immunization, blood samples were collected from the tail vein to collect serum. All mice were challenged with a lethal dose of *S. aureus* USA300 (4 × 10^8^ CFU/mouse) via tail vein injection. Survival was monitored daily for 5 days post-challenge.

#### 2.9.3. ELISA Assays

HI antigen-specific IgG, IgG1, IgG2a, and IgG2b were quantitatively detected. The brief procedure was as follows: A 2 μg/mL HI antigen solution was added to a 96-well plate and incubated overnight at 4 °C. The plate was washed with PBST solution and then blocked with blocking buffer for 2 h, followed by another wash with PBST solution. Subsequently, serum samples were added to the 96-well plate, serially diluted, and incubated for another 1 h. After washing the 96-well plate with PBST solution, 100 μL per well of horseradish peroxidase-conjugated goat anti-mouse IgG, IgG1, IgG2a, and IgG2b (Bethyl Biotech) were added and incubated at 37 °C for 1 h. Next, 100 μL per well of the corresponding enzyme substrate (Tian Gen) was added and allowed to react in a 37 °C incubator for 10 min. Finally, 50 μL per well of 2 M H_2_SO_4_ was added to stop.

### 2.10. Statistics

All statistical analyses were performed using GraphPad Prism software 10.1.2. ELISA data were analyzed by one-way ANOVA followed by Tukey’s post hoc test for multiple comparisons, while survival data were assessed using the Mantel–Cox (log-rank) test. Results were expressed as mean ± standard deviation (SD), and statistical significance was set at *p* < 0.05.

## 3. Results

### 3.1. Potential Target Genes of Epimedium and Immunomodulation

Bioactive compounds from Epimedium were initially screened from the TCMSP database (https://tcmsp-e.com/, accessed on 2 January 2026) using thresholds of oral bioavailability (OB ≥ 30%) and drug-likeness (DL ≥ 0.18), as previously described. This initial screening identified 23 compounds. After applying SwissADME filtering to the remaining compounds, a total of 16 compounds (excluding ICA) were retained. Combined with the manually reinstated ICA, a final set of 17 compounds (Table 1) was used for target prediction. These compounds are: Magnograndiolide, olivil, 6-hydroxy-11,12-dimethoxy-2,2-dimethyl-1,8-dioxo-2,3,4,8-tetrahydro-1H-Isochromeno [3,4-h] isoquinolin-2-ium, Yinyanghuo A,1,2-bis(4-hydroxy-3-methoxyphenyl) propan-1,3-diol, Icariin, Yinyanghuo E, 8-(3-methylbut-2-enyl)-2-phenyl-chromone, quercetin, Yinyanghuo C, Anhydroicaritin, kaempferol, C-Homoerythrinan, 1,6-didehydro-3,15,16-trimethoxy-, (3.beta.)-8-Isopentenyl-kaempferol, luteolin, Chryseriol, DFV. Subsequently, prediction of the potential target genes for these compounds was performed with the SwissTargetPrediction platform. After the removal of duplicate entries, the screening yielded 488 Epimedium-associated predicted targets.

A search for the keyword “immunomodulation” in the GeneCards and OMIM databases yielded 1425 and 24 related genes, respectively. After the removal of duplicate entries, a total of 1442 immunomodulation-related genes were identified.

### 3.2. PPI Network and Core Targets

**Figure 2:**
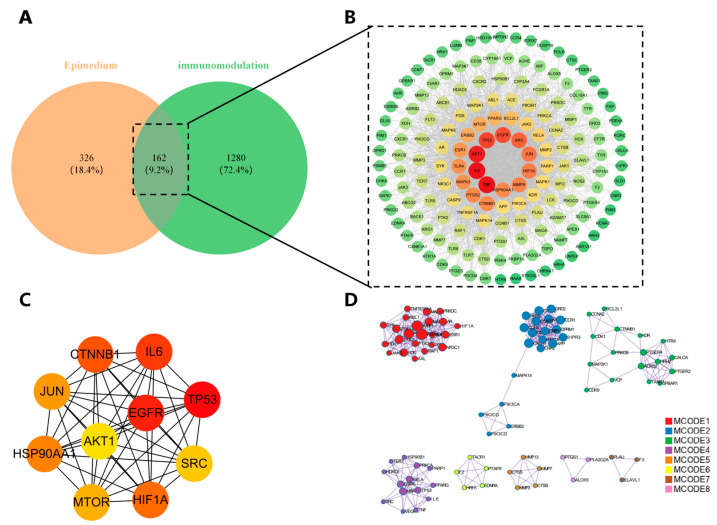
Identification of Core Targets and Network Modules Underlying the Immunomodulatory Activity of Epimedium (**A**) Venn diagram showing the intersection between 488 Epimedium-associated predicted targets and 1442 immunomodulation-associated targets, yielding 162 overlapping targets. (**B**) PPI network of the 162 intersecting targets, constructed using the STRING database (confidence score > 0.4) and visualized with Cytoscape 3.10.3. Nodes represent proteins; edges represent interactions. Node size reflects degree centrality. (**C**) Network representation of the ten principal hub genes, as determined by the CytoHubba plugin, with node size and color indicating their importance in the network. (**D**) Molecular complexes detected by the MCODE algorithm, revealing 8 densely connected clusters within the PPI network, suggesting potential functional modules.

A total of 162 overlapping genes were identified at the intersection of Epimedium and immunomodulation targets. These were established as the core targets for this study. (Figure 2A). We imported these overlapping targets into the STRING database for PPI network analysis. Cytoscape 3.10.3 was employed to visualize the generated PPI network to construct a comprehensive interaction map (Figure 2B), and are considered hub genes of Epimedium and immunomodulation. Subsequently, we used the CytoHubba plugin to identify the top 10 core genes via the Degree method. These hub genes were identified as IL6, TP53, EGFR, CTNNB1, HIF1A, HSP90AA1, JUN, MTOR, SRC, AKT1 (Figure 2C). Furthermore, to pinpoint densely connected protein clusters within the target network, we employed the Molecular Complex Detection (MCODE) algorithm. The analysis revealed a total of eight significant clusters (Figure 2D).

### 3.3. KEGG Pathway and GO Functional Enrichment Analyses

**Figure 3:**
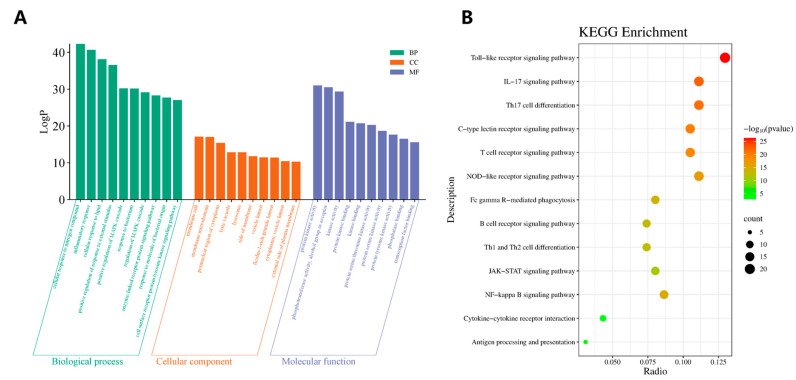
Functional enrichment analysis of intersecting targets. (**A**) Bar chart of the top 10 GO enrichment terms for BP, CC, and MF. (**B**) Bubble plot of the 13 enriched KEGG pathways. The x-axis represents the gene ratio, the horizontal axis denotes the gene ratio, while the vertical axis displays the names of the pathways. The size of each point corresponds to the count of enriched genes, and its color reflects the adjusted *p*-value.

The Metascape platform was utilized to perform KEGG pathway and GO functional enrichment analyses on the 162 intersecting targets. The GO functional enrichment analysis yielded 1791 Biological Process (BP), 125 Cellular Component (CC), and 182 Molecular Function (MF). The targets were significantly enriched in several key areas: for BP, these included nitrogen compounds, inflammatory response, and cellular response to lipids; for CC, included membrane rafts, membrane microdomains, and the perinuclear region of the cytoplasm; and for MF, included protein kinase activity, phosphotransferase activity with an alcohol group as acceptor, and kinase activity. Based on *p*-value ranking, the top ten enriched GO terms from each category (BP, CC, MF) were selected and visualized using the Bioinformatics platform (Figure 3A).

A total of 198 significant pathways were identified through the KEGG pathway analysis. In the KEGG network visualization, the size of each node is proportional to the number of target genes enriched in the corresponding pathway. Node color corresponds to the associated *p*-value, transitioning from green (higher *p*-value) to red (lower *p*-value). Based on the enrichment results, 13 immunomodulation pathways were selected to generate a bubble plot. These pathways include the Toll-like receptor, IL-17, Th17 cell differentiation, C-type lectin receptor, T cell receptor, NOD-like receptor, NF-kappa B, Fc gamma R-mediated phagocytosis, B cell receptor, Th1 and Th2 cell differentiation, JAK-STAT, Antigen processing and presentation, and Cytokine-cytokine receptor interaction signaling pathway (Figure 3B).

### 3.4. “Epimedium-Target-Pathway-Immunomodulation” Network

**Figure 4:**
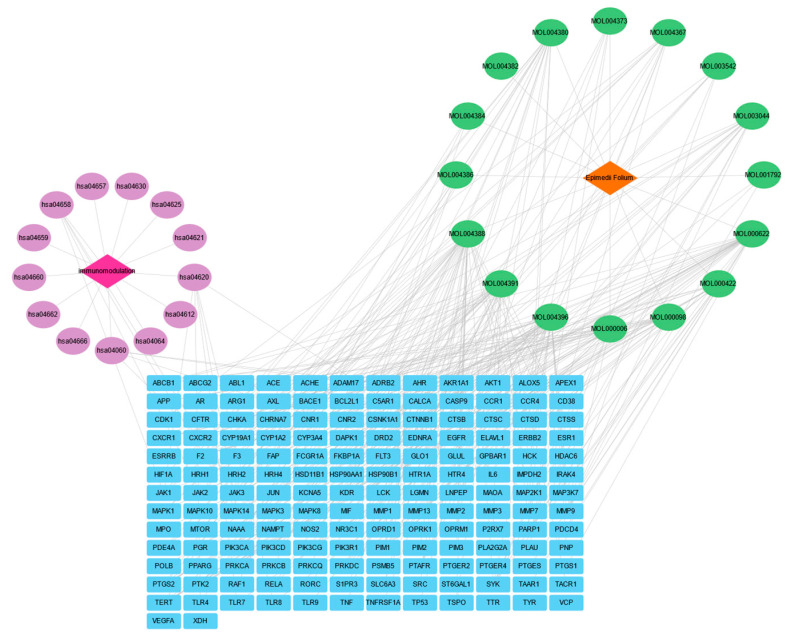
The “Epimedium-Target-Pathway-Immunomodulation” Network. The network comprises 189 nodes and 632 edges, showing the complex interactions between the bioactive compounds of Epimedium, core targets, key signaling pathways, and Immunomodulation. Orange diamonds represent the drug (Epimedium), fuchsia diamonds represent the disease (immunomodulation), green ovals represent bioactive compounds, purple ovals represent signaling pathways, and blue rectangles represent core protein targets. The gray lines indicate the interconnections among these bioactive compounds.

The “Epimedium-Target-Pathway-Immunomodulation” network illustrating the relationship between Epimedium and immunomodulation is presented in Figure 4.

This network comprises 189 nodes and 632 edges, demonstrating the intricate interconnections among the bioactive compounds of Epimedium, core targets, relevant signaling pathways, and immunomodulation. In the visualization, red diamond-shaped nodes represent the Epimedium drug, while fuchsia diamond-shaped nodes represent immunomodulation. Green oval nodes denote the bioactive compounds, purple oval nodes signify signaling pathways, and blue rectangular nodes indicate core protein targets. Within the network, Epimedium bioactive compounds, targets, pathways, and immunomodulation are linked by gray lines, indicating their interrelationships. This comprehensive network suggests that Epimedium participates in the immunoregulatory process by acting on multiple targets across various pathways.

### 3.5. Molecular Docking and Visualization

The intersection of the top ten genes ranked by Degree and the top ten ranked by MCC yielded eight core genes: CTNNB1, AKT1, EGFR, HIF1A, IL6, JUN, SRC, and TP53. Among these, HIF1A and SRC were excluded from subsequent analysis because their molecular docking binding energies were close to or equal to 0 kcal/mol, suggesting poor binding affinity. The remaining six genes were selected for molecular docking.

Generally, a binding energy of <0 kcal/mol suggests that the ligand-target binding occurs spontaneously. An energy < −4.25 kcal/mol is considered to represent good docking affinity, while an energy < −7.0 kcal/mol is indicative of strong docking affinity. However, it is important to note that docking scores are predictive and do not directly equate to functional biological activity.

**Figure 5:**
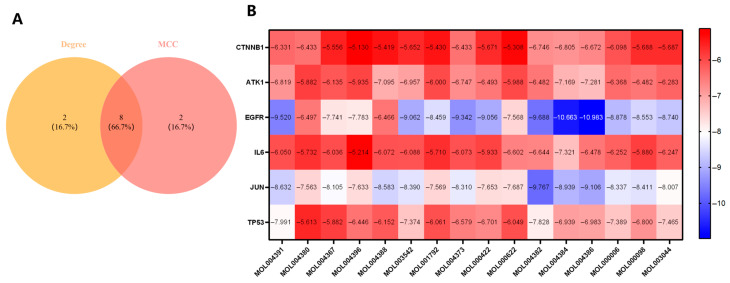
Screening of core targets and validation of binding affinity through molecular docking. (**A**) Venn Diagram of the Intersection between Top 10 Degree-and MCC-Ranked Genes. (**B**) Binding affinities of 17 Epimedium bioactive compounds with six core targets are visualized in a heatmap. A color gradient from blue to red represents the range of binding energies, with blue indicating stronger affinity (lower energy) and red indicating weaker affinity (higher energy). The exact numerical values are annotated within each cell.

Molecular docking analysis was subsequently performed between the 17 primary bioactive compounds of Epimedium (DFV, Chryseriol, 8-Isopentenyl-kaempferol, Kaempferol, Olivil, Anhydroicaritin, C-Homoerythrinan, 1,6-didehydro-3,15,16-trimethoxy-, (3.beta.)-, Yinyanghuo A, Yinyanghuo C, Yinyanghuo E, 6-hydroxy-11,12-dimethoxy-2,2-dimethyl-1,8-dioxo-2,3,4,8-tetrahydro-1H-isochromeno[3,4-h] isoquinolin-2-ium, 8-(3-methylbut-2-enyl)-2-phenyl-chromone, 1,2-bis(4-hydroxy-3-methoxy-phenyl) propan-1,3-diol, Luteolin, Magnograndiolide, and Quercetin) and the six core targets (CTNNB1, AKT1, EGFR, IL6, JUN, TP53) (Figure 5).

**Figure 6:**
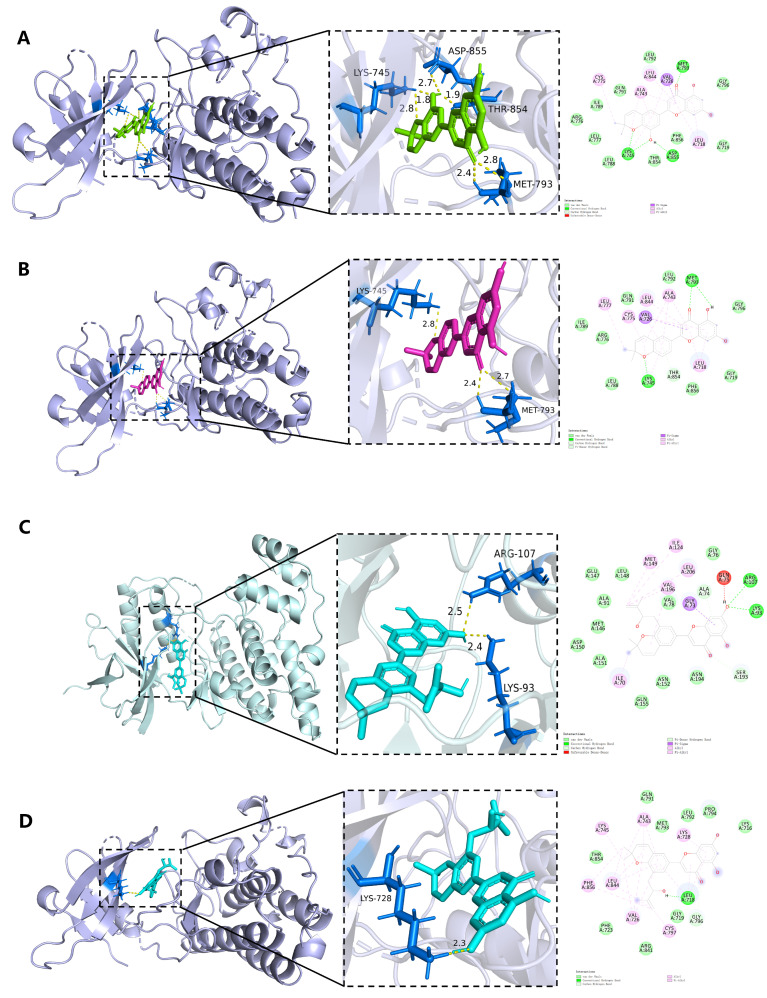

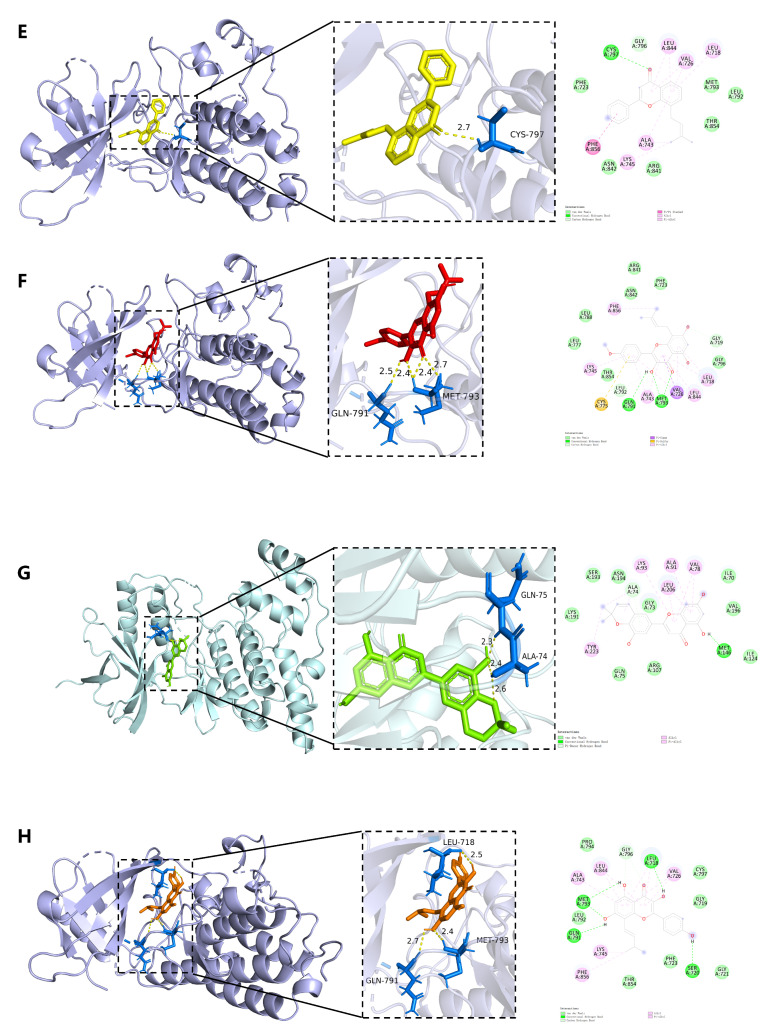
The 3D, 2D, and hydrogen-bonding interaction diagrams illustrate the binding of Epimedium bioactive compounds to hub genes. In these visualizations, hydrogen bonds are depicted as yellow dashed lines, with their corresponding bond lengths labeled accordingly. (**A**) Yinyanghuo E-EGFR. (**B**) Yinyanghuo C-EGFR. (**C**) Yinyanghuo A-JUN. (**D**) Yinyanghuo A-EGFR. (**E**) 8-(3-methylbut-2-enyl)-2-phenyl-chromone-EGFR. (**F**) Anhydroicaritin-EGFR. (**G**) Yinyanghuo E-JUN. (**H**) 8-Isopentenyl-kaempferol-EGFR.

We present the binding affinities and detailed molecular docking results for the bioactive compounds and hub targets. The results revealed that Yinyanghuo E exhibited the lowest binding energy with EGFR, indicating the strongest binding capability. The eight complex conformations with optimal binding energies were identified for subsequent visualization and analysis using PyMOL to generate 3D and 2D interaction diagrams (Figure 6). These top complexes were: Yinyanghuo E-EGFR, Yinyanghuo C-EGFR, Yinyanghuo A-JUN, Yinyanghuo A-EGFR, 8-(3-methylbut-2-enyl)-2-phenyl-chromone-EGFR, Anhydroicaritin-EGFR, Yinyanghuo E-JUN, and 8-Isopentenyl-kaempferol-EGFR.

The analysis revealed that these complexes formed stable interactions, with the number of hydrogen bonds formed as follows: Yinyanghuo E-EGFR (6), Yinyanghuo C-EGFR (3), Yinyanghuo A-JUN (2), Yinyanghuo A-EGFR (1), 8-(3-methylbut-2-enyl)-phenyl-chromone-EGFR (1), Anhydroicaritin-EGFR (4), Yinyanghuo E-JUN (3), and 8-Isopentenyl-kaempferol-EGFR.

### 3.6. MD Simulation

To evaluate the binding stability of the identified ligand–receptor complexes, MD simulations were performed for the following systems: Yinyanghuo E–EGFR, Yinyanghuo C–EGFR, Yinyanghuo A–JUN, Yinyanghuo A–EGFR, 8-(3-methylbut-2-enyl)-2-phenyl-chromone–EGFR, Anhydroicaritin–EGFR, Yinyanghuo E–JUN, and 8-Isopentenyl-kaempferol–EGFR. The conformational stability, structural compactness, solvent accessibility, hydrogen-bond interactions, residue flexibility, and free-energy landscapes of each complex were systematically analyzed.

#### 3.6.1. MD Simulation Yinyanghuo E and EGFR Complexes

**Figure 7:**
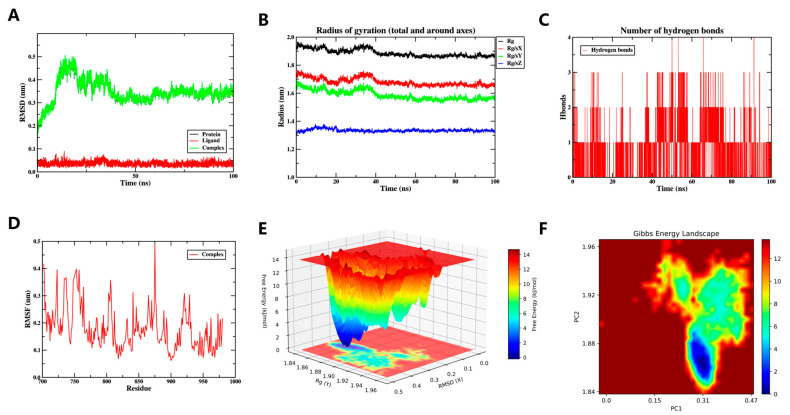
The MD simulation of the Yinyanghuo E-EGFR complex over 100 ns. (**A**) The RMSD plot of the Yinyanghuo E-EGFR complex, showing stabilization after 50 ns within 0.3–0.4 nm, indicating conformational stability. (**B**) The Rg plot of the Yinyanghuo E-EGFR complex, reflecting protein compactness, stable values suggest no major unfolding. (**C**) The count of hydrogen bonds in the Yinyanghuo E-EGFR complex. (**D**) The RMSF plot of the Yinyanghuo E-EGFR complex. (**E**) The 3D Gibbs energy landscape of the Yinyanghuo E-EGFR complex. (**F**) The 2D Gibbs energy landscape of the Yinyanghuo E-EGFR complex. Deep blue basins (Rg 1.84–1.86 nm, RMSD 0.3–0.4 nm) represent global free energy minima, indicating thermodynamically favorable binding.

Root Mean Square Deviation (RMSD) serves as a standard metric for assessing the conformational stability of proteins and ligands. It quantifies positional shifts in atoms relative to their starting coordinates, with smaller deviations reflecting greater structural steadiness. Accordingly, the equilibrium state of the simulation was assessed through RMSD analysis. As illustrated in Figure 7A, the system stabilized after 50 ns, with subsequent fluctuations maintained within a narrow range of 0.3 to 0.4 nm. These results suggest that the small molecule remains highly stable while complexed with the target protein.

The Radius of Gyration (Rg) quantifies protein compactness, thereby providing insight into global structural changes. A significant shift in Rg values typically reflects a notable expansion of the overall system. Throughout the simulation, the complex exhibited stable yet fluctuating Rg values, reflecting conformational dynamics of the ligand-protein system. (Figure 7B).

Calculation of the Solvent Accessible Surface Area (SASA) for the protein structure remained stable upon ligand binding (Supplementary Figure S1). Taken together, these results indicate that the global protein conformation is not substantially perturbed by ligand binding.

The formation of hydrogen bonds significantly contributes to ligand–protein binding. The number of ligand–protein hydrogen bonds as a function of time is shown in Figure 7C. The hydrogen bond count ranged from 0 to 4, with one hydrogen bond being the most common interaction pattern observed. The persistent hydrogen bonds are consistent with a favorable ligand-protein interaction.

Root Mean Square Fluctuation (RMSF) quantifies the flexibility of individual amino acid residues in a protein. As shown in Figure 7D, low RMSF values (primarily <0.4 nm) were observed for the complex, reflecting a rigid and stable conformation. In summary, the system exhibits stable binding with favorable hydrogen-bond interactions, confirming effective ligand-protein engagement.

To comprehensively investigate the dynamic conformational stability and underlying interaction mechanisms of the protein–ligand system, two-dimensional (2D) (Figure 7E) and three-dimensional (3D) (Figure 7F) Free Energy Landscape (FEL) analyses were conducted. These landscapes quantitatively characterize the free energy changes during conformational evolution using Rg and RMSD as reaction coordinates. The analysis revealed a global free energy minimum region (indicated by deep blue basins in the figures) within specific ranges of Rg (1.84–1.86 nm) and RMSD (0.3–0.4 nm), suggesting that the system achieves optimal thermodynamic stability in this conformational state.

Collectively, the stable binding of Yinyanghuo E to EGFR throughout the 100 ns simulation suggests a favorable interaction at the molecular level. However, this computational observation does not confirm biological activity; it provides a hypothesis for future experimental testing (e.g., surface plasmon resonance or cellular assays).

#### 3.6.2. MD Simulation Yinyanghuo C and EGFR Complexes

**Figure 8:**
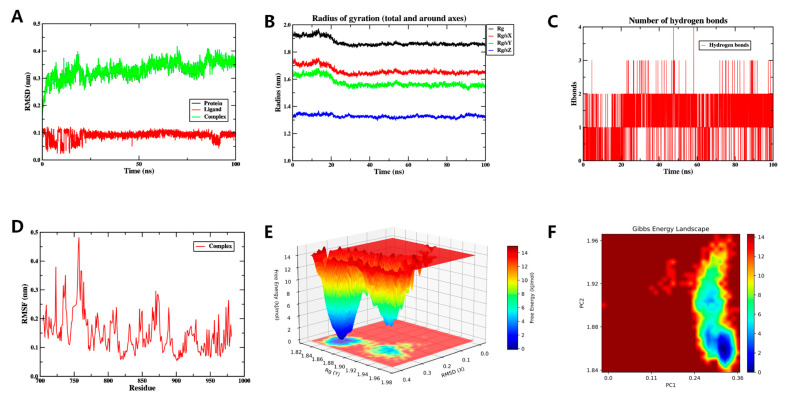
The MD simulation of the Yinyanghuo C-EGFR over 100 ns. (**A**) The RMSD plot of the Yinyanghuo C-EGFR complex, showing stabilization after 25 ns within 0.25–0.35 nm, indicating conformational stability. (**B**) The Rg plot of the Yinyanghuo C-EGFR complex, reflecting protein compactness; stable values suggest no major unfolding. (**C**) The count of hydrogen bonds in the Yinyanghuo C-EGFR complex. (**D**) The RMSF plot of the Yinyanghuo C-EGFR complex. (**E**) The 3D Gibbs energy landscape of the Yinyanghuo C-EGFR complex. (**F**) The 2D Gibbs energy landscape of the Yinyanghuo C-EGFR complex. Deep blue basins (Rg 1.84–1.86 nm, RMSD 0.24–0.36 nm) represent global free energy minima, indicating thermodynamically favorable binding.

The RMSD stabilized within 0.25–0.35 nm after 25 ns (Figure 8A). Rg varied slightly during the simulation (Figure 8B), while SASA remained largely unchanged (Supplementary Figure S2). An average of two hydrogen bonds (range 0–5) was maintained between the ligand and EGFR (Figure 8C). RMSF values were predominantly below 0.4 nm (Figure 8D). FEL analysis indicated a low-energy basin at Rg = 1.84–1.86 nm and RMSD = 0.24–0.36 nm (Figure 8E,F).

Collectively, stable binding of Yinyanghuo C to EGFR throughout the simulations suggests it may confer relevant biological activity.

### 3.7. Effect of ICA Adjuvant on HI-Specific Antibody Responses and Protective Efficacy Against S. aureus USA300 Challenge

**Figure 9:**
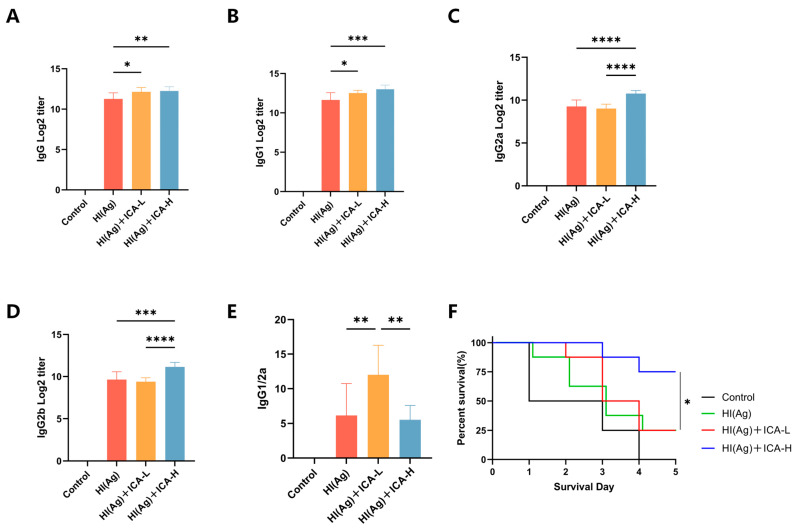
Effects of ICA on HI-specific antibody levels and protection against *S. aureus* USA300 challenge in mice. (**A**–**E**) HI-specific IgG, IgG1, IgG2a, IgG2b, and the IgG1/IgG2a ratio in serum were measured by ELISA. (**F**) Survival rates of mice challenged with USA300 were monitored daily for 5 days. Data are presented as mean ± SD (*n* = 8 per group). * *p* < 0.05, ** *p* < 0.01, *** *p* < 0.001, **** *p* < 0.0001.

To evaluate the adjuvant efficacy of ICA in immunized mice, humoral immune responses were assessed by measuring the levels of IgG, IgG1, IgG2a, and IgG2b, as well as the IgG1/IgG2a ratio, in serum collected and isolated at 7 days post-booster immunization (Figure 9A–E and Supplementary Figure S9). Compared with the HI group, the HI(Ag)+ICA-H group exhibited significantly increased levels of IgG, IgG1, IgG2a and IgG2b. Furthermore, the IgG1/IgG2a ratio in ICA-immunized mice was greater than 1, indicating a Th2-biased immune response. To evaluate the functional relevance of the antibody responses, a challenge experiment was performed using *S. aureus* USA300. As shown in Figure 9F, all mice in the control group died within 5 days post-challenge (0% survival). In contrast, the high-dose ICA group exhibited the strongest protective effect, with significantly higher survival rates than the HI group. These data demonstrate that HI(Ag)+ICA-H enhances HI antigen-specific antibody levels in immunized mice.

## 4. Discussion

Using an integrated strategy of network pharmacology, molecular docking, and MD simulations combined with in vivo experimental validation, this study provides predictive and initial experimental evidence for the adjuvant potential of Epimedium. The computational analyses identified 17 bioactive compounds, 488 Epimedium-associated predicted targets, and 13 immunomodulatory pathways. The in vivo experiments demonstrated that ICA, as a representative component, significantly enhanced HI-specific antibody responses and improved survival against the lethal *S. aureus* USA300 challenge. However, it is critical to emphasize that the proposed molecular mechanisms—such as modulation of IL6, EGFR, or AKT1—remain computational hypotheses that have not been experimentally verified.

Our network pharmacology analysis predicted IL6, TP53, EGFR, AKT1, and JUN as core targets of Epimedium in immunomodulation. These targets have documented associations with immune processes. IL6 is a pleiotropic cytokine that promotes B-cell differentiation into antibody-secreting plasma cells and supports the development of T follicular helper (Tfh) cells [51]. AKT1, a central node in the PI3K-Akt signaling pathway, regulates dendritic cell maturation and MHC-II expression, thereby influencing antigen presentation efficiency [52]. EGFR and JUN are involved in the activation of the AP-1 transcription factor, which controls the expression of cytokines (e.g., IL-2, IFN-γ) that are critical for T-cell proliferation and effector function [53,54]. Nevertheless, it is important to emphasize that these mechanistic links are predictions derived solely from computational analyses. The present study did not perform gene knockdown, protein expression, or pathway activation assays to confirm that these targets mediate the adjuvant effects of ICA. Therefore, these predictions should be considered hypothesis-generating and warrant future experimental investigation.

Our experimental results showed that ICA induced an IgG1/IgG2a ratio greater than 1, indicating a Th2-biased humoral response. This immune profile is particularly relevant for vaccines targeting extracellular bacterial pathogens such as *S. aureus*. The primary protective mechanism against *S. aureus* is opsonophagocytic killing, mediated by antibodies that enhance bacterial uptake and destruction by phagocytes. In mice, both IgG1 and IgG2b are capable of activating complement and promoting opsonophagocytosis. Notably, our ICA-adjuvanted group also exhibited elevated levels of HI-specific IgG2b (Figure 9D). The higher IgG2b response may further contribute to protective efficacy, as IgG2b has been shown to bind to Fcγ receptors on phagocytes and facilitate bacterial clearance [55]. Th2-skewed responses promote B-cell activation and antibody production, which are functionally advantageous in this context [56]. Indeed, ICA-adjuvanted mice showed significantly improved survival after lethal USA300 challenge, confirming that the induced antibody repertoire was sufficient for protection. For intracellular pathogens (e.g., Mycobacterium tuberculosis or viruses), a Th1-biased adjuvant would be preferred. Therefore, the value of ICA as an adjuvant should be considered in the context of the target pathogen and the desired immune outcome. Future studies should evaluate ICA’s adjuvant activity with other antigens (e.g., viral subunit vaccines) to determine whether its Th2 bias is conserved or antigen-dependent.

Several limitations of this study should be considered when interpreting the findings. First, our experimental validation focused exclusively on humoral immune responses (serum IgG subclasses and protective efficacy). We did not measure T-cell responses, including CD4^+^ and CD8^+^ T-cell proliferation, intracellular cytokine production (IFN-γ, IL-4, IL-17A), Tfh cell frequency, or cytotoxic T lymphocyte activity. Although KEGG enrichment analysis predicted the involvement of T-cell receptor signaling and Th17/Th1/Th2 differentiation pathways, these predictions remain unvalidated. Consequently, our conclusions are limited to antibody-mediated protection, and the proposed T-cell mechanisms require future experimental testing. Second, while our computational analysis included multiple Epimedium bioactive compounds, the in vivo validation used only ICA. Although ICA is the major quality control component and a well-documented immunomodulatory agent, other bioactive compounds (e.g., Anhydroicaritin, yinyanghuo E) may contribute synergistically or independently to the adjuvant effect. Future studies should evaluate Epimedium extracts or compound combinations. Third, the core targets predicted by network pharmacology (IL6, TP53, EGFR, AKT1, etc.) were not experimentally verified. No gene-expression, protein-expression, or pathway-activation assays were performed. Therefore, the mechanistic claims are purely predictive. Fourth, the protective effect observed with ICA was demonstrated only in the *S. aureus* USA300 challenge model. Whether ICA acts as an adjuvant for other antigens (e.g., viral or tumor antigens) remains unknown.

In summary, this study provides computational predictions and initial experimental evidence that Epimedium, through its representative component ICA, may function as a vaccine adjuvant by modulating multiple immunomodulatory targets and pathways. The Th2-biased antibody response induced by ICA was sufficient to confer protection against a lethal *S. aureus* challenge. However, the proposed molecular mechanisms (including the involvement of IL6, AKT1, EGFR, and T-cell pathways) remain hypotheses that require experimental validation. Future research should focus on (i) verifying target engagement using biochemical assays, (ii) assessing T-cell responses and APC activation, (iii) comparing ICA with licensed adjuvants, and (iv) evaluating its adjuvant activity with other vaccine antigens. These efforts will help determine whether ICA or Epimedium extracts can be developed into safe and effective vaccine adjuvants.

## 5. Conclusions

This study suggests that Epimedium and its bioactive compounds may function as promising vaccine adjuvants through multi-target modulation of key immune signaling pathways, as revealed by integrating network pharmacology, molecular docking, MD simulations, and experimental validation. The stable binding of core bioactive compounds to immunoregulatory targets, supported by dynamic simulations, provides computational evidence for their potential adjuvant activity. Therefore, the proposed mechanisms should be considered hypotheses requiring further experimental confirmation. Future research should focus on elucidating the specific intracellular signal transduction mechanisms involved and conducting formulation optimization studies, with the ultimate goal of developing Epimedium into a highly effective and safe practical vaccine adjuvant.

## Figures and Tables

**Table 1:** Basic information of the 17 potential bioactive compounds in Epimedium.

Number	Mol ID	Molecular Name	OB (%)	DL	Molecular Structure
1	MOL000622	Magnograndiolide	63.71	0.19	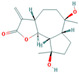
2	MOL004367	olivil	62.23	0.41	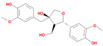
3	MOL004388	6-hydroxy-11,12-dimethoxy-2,2-dimethyl-1,8-dioxo-2,3,4,8-tetrahydro-1H-isochromeno[3,4-h] isoquinolin-2-ium	60.64	0.66	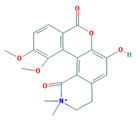
4	MOL004382	Yinyanghuo A	56.96	0.77	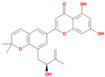
5	MOL004396	1,2-bis(4-hydroxy-3-methoxyphenyl) propan-1,3-diol	52.31	0.22	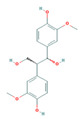
6	MOL004425	Icariin	41.58	0.61	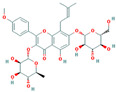
7	MOL004386	Yinyanghuo E	51.63	0.55	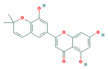
8	MOL004391	8-(3-methylbut-2-enyl)-2-phenyl-chromone	48.54	0.25	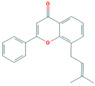
9	MOL000098	quercetin	46.43	0.28	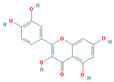
10	MOL004384	Yinyanghuo C	45.67	0.5	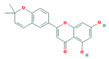
11	MOL004373	Anhydroicaritin	45.41	0.44	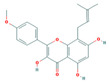
12	MOL000422	kaempferol	41.88	0.24	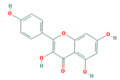
13	MOL004380	C-Homoerythrinan,1,6-didehydro-3,15,16-trimethoxy-, (3.beta.)-	39.14	0.49	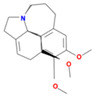
14	MOL003542	8-Isopentenyl-kaempferol	38.04	0.39	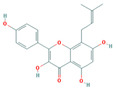
15	MOL000006	luteolin	36.16	0.25	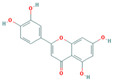
16	MOL003044	Chryseriol	35.85	0.27	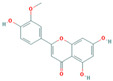
17	MOL001792	DFV	32.76	0.18	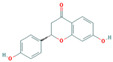

## Data Availability

The original contributions presented in this study are included in the article/Supplementary Material. Further inquiries can be directed to the corresponding authors.

## Supplementary Materials

The following supporting information can be downloaded at: https://www.mdpi.com/article/10.3390/vaccines14050385/s1, Figure S1: SASA of the MD simulation of the Yinyanghuo C-EGFR complex; Figure S2: SASA of the MD simulation of the Yinyanghuo C-EGFR complex; Figure S3: The MD simulation of the Yinyanghuo A-JUN; Figure S4: The MD simulation of the Yinyanghuo A-EGFR; Figure S5: The MD simulation of the 8-(3-methylbut-2-enyl)-2-phenyl-chromone-EGFR; Figure S6: The MD simulation of the Anhydroicaritin-EGFR; Figure S7: The MD simulation of the Yinyanghuo E-JUN; Figure S8: The MD simulation of the 8-Isopentenyl-kaempferol-EGFR. Figure S9: Effect of ICA adjuvant on HI-specific antibody responses.
