# Supplementary material for: Exploring the Vaccine Adjuvant Effect and Mechanism of Epimedium Using Network Pharmacology, Molecular Docking, and Molecular Dynamics Simulations
Source: Vaccines (Basel). 2026 Apr 26;14(5):385. doi: 10.3390/vaccines14050385 (PMC13211340; doi:10.3390/vaccines14050385)
Supplement: Supplementary file 1 [file vaccines-14-00385-s001.zip › vaccines-4232755-supplementary.pdf]

Supplementary Information

Exploring the Vaccine Adjuvant Effect and Mechanism of Epimedium Using Network Pharmacology, Molecular Docking, and Molecular Dynamics Simulations

1.SASA of MD simulation of the Yinyanghuo E-EGFR complex

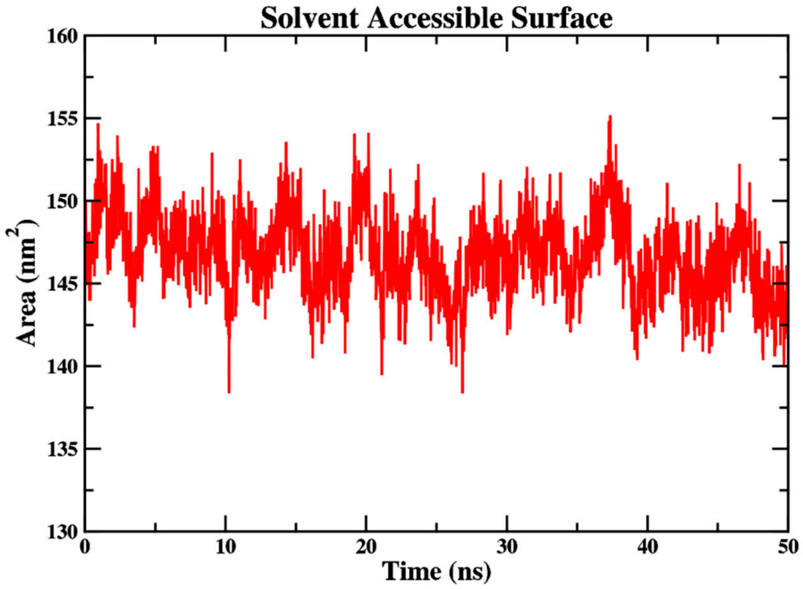

Supplement Figure S1. SASA of the MD simulation of the Yinyanghuo C-EGFR complex.

2.*SASA of* MD simulation of the Yinyanghuo C-EGFR complex.

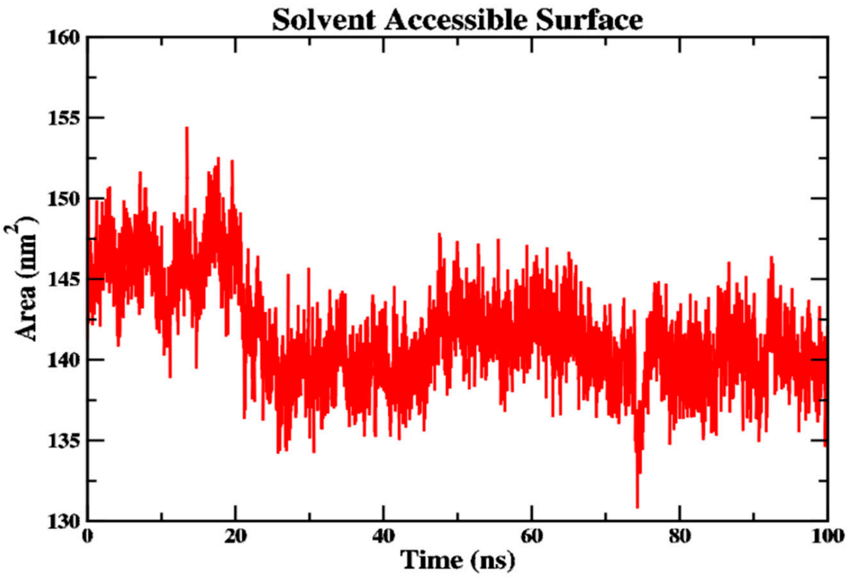

Supplement Figure S2. SASA of the MD simulation of the Yinyanghuo C-EGFR complex.

3. MD Simulation Yinyanghuo A and JUN Complexes

The system equilibrated after 60 ns, with RMSD fluctuating between 0.25–0.35 nm (Supplement Figure S3A). Minor Rg variations were observed (Supplement Figure S3B), and SASA showed no marked alteration (Supplementary Figure S3G). Hydrogen bonding ranged from zero to three, averaging one bond (Supplement Figure S3C). Residue flexibility was low (RMSF <0.5 nm; Supplement Figure S3D). The FEL displayed an energy minimum at Rg = 1.73–1.78 nm and RMSD = 0.25–0.38 nm (Supplement Figure S3E,3F)

Collectively, these data indicate that the binding between Yinyanghuo A and JUN remains stable throughout the molecular dynamics simulations and may possess potential biological activity.

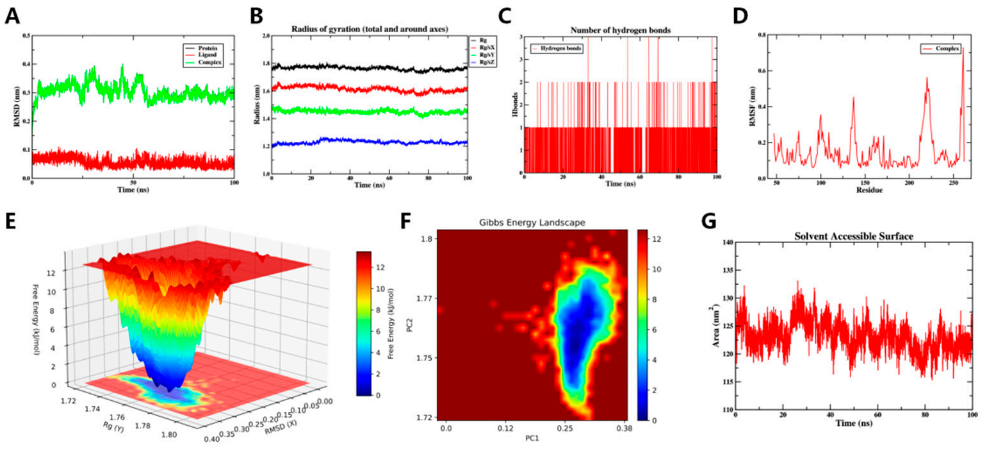

Supplement Figure S3. The MD simulation of the Yinyanghuo A-JUN. (A) The RMSD plot of the Yinyanghuo A-JUN complex. (B) The Rg plot of the Yinyanghuo A-JUN complex. (C)The number of hydrogen bonds in the Yinyanghuo A-JUN complex. (D) The RMSF plot of the Yinyanghuo A-JUN complex. (E) The 3D Gibbs energy landscape of the Yinyanghuo A-JUN complex. (F) The 2D Gibbs energy landscape of the Yinyanghuo A-JUN complex. (G) The SASA of the Yinyanghuo A-JUN complex.

4. MD Simulation Yinyanghuo A and EGFR Complexes

RMSD reached equilibrium within 25 ns, varying between 0.25–0.4 nm (Supplement Figure S4A). Rg fluctuated moderately (Supplement Figure S4B), and SASA was not significantly affected (Supplementary Figure S4G). Approximately two hydrogen bonds (range 0–4) were sustained on average (Supplement Figure S4C). RMSF values were below 0.4 nm for most residues (Supplement Figure S4D). The free-energy minimum was located at Rg = 1.82–1.86 nm and RMSD = 0.24–0.36 nm (Supplement Figure S4E,F).

Collectively, these data indicate that the binding between Yinyanghuo A and EGFR remains stable throughout the molecular dynamics simulations and may possess potential biological activity.

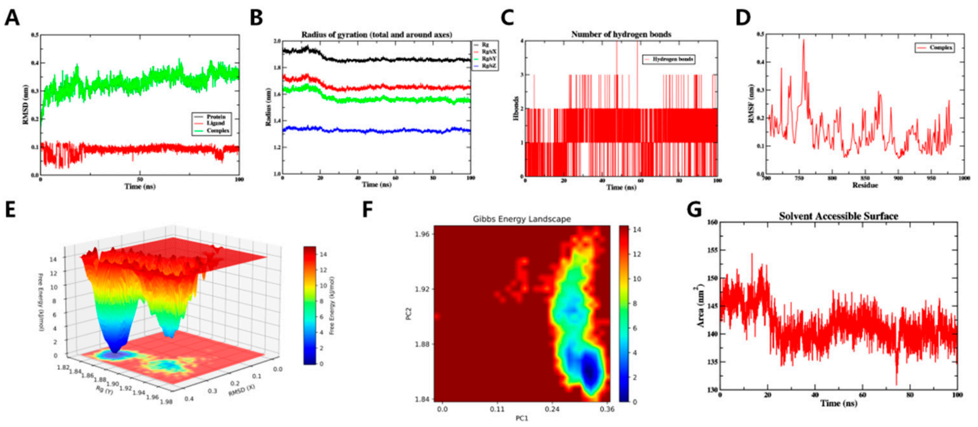

Supplement Figure S4. The MD simulation of the Yinyanghuo A-EGFR. (A) The RMSD plot of the Yinyanghuo A-EGFR complex. (B) The Rg plot of the Yinyanghuo A-EGFR complex. (C) The number of hydrogen bonds in theYinyanghuo A-EGFR complex. (D) The RMSF plot of the Yinyanghuo A-EGFR complex. (E) The 3D Gibbs energy landscape of the Yinyanghuo A-EGFR complex. (F) The 2D Gibbs energy landscape of the Yinyanghuo A-EGFR complex. (G) The SASA of the Yinyanghuo A- EGFR complex.

5. MD Simulation 8-(3-methylbut-2-enyl)-2-phenyl-chromone and EGFR Complexes

After 50 ns, RMSD values stabilized in the range of 0.3–0.4 nm (Supplement Figure S5A). Rg exhibited small fluctuations (Supplement Figure S5B), and SASA remained consistent (Supplementary Figure S5G). Hydrogen-bond counts ranged from zero to five, with an average of two (Supplementary Figure S5C). RMSF analysis indicated low flexibility (mostly <0.4 nm; Supplementary Figure S5D). FEL profiles showed an energy basin at Rg = 1.72–1.78 nm and RMSD = 0.20–0.35 nm (Supplementary Figure S5E,F).

Collectively, these data indicate that the binding between 8-(3-methylbut-2-enyl)-2-phenyl-chromone and EGFR remains stable throughout the molecular dynamics simulations and may possess potential biological activity.

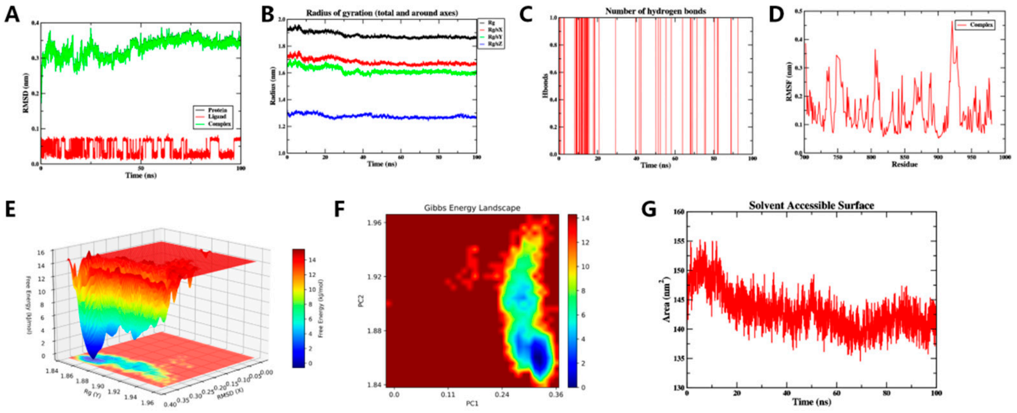

Supplementary Figure S5. The MD simulation of the 8-(3-methylbut-2-enyl)-2-phenyl-chromone-EGFR. (A) The RMSD plot of the 8-(3-methylbut-2-enyl)-2-phenyl-chromone-EGFR complex. (B) The Rg plot of the 8-(3-methylbut-2-enyl)-2-phenyl-chromone-EGFR complex. (C) The number of hydrogen bonds in the 8-(3-methylbut-2-enyl)-2-phenyl-chromone-EGFR complex. (D) The RMSF plot of the 8-(3-methylbut-2-enyl)-2-phenyl-chromone-EGFR complex. (E) The 3D Gibbs energy landscape of the 8-(3-methylbut-2-enyl)-2-phenyl-chromone-EGFR complex. (F) The 2D Gibbs energy landscape of the

8-(3-methylbut-2-enyl)-2-phenyl-chromone-EGFR complex. (G) The SASA of the 8-(3-methylbut-2-enyl)-2-phenyl-chromone-EGFR complex.

6. MD Simulation Icaritin and EGFR Complexes

The RMSD stabilized after 40 ns within 0.3–0.4 nm (Supplementary Figure S6A). Rg varied slightly (Supplementary Figure S6B), and SASA changes were negligible (Supplementary Figure S6G). An average of one hydrogen bond (range 0–2) was maintained (Supplementary Figure S6C). RMSF values were generally below 0.4 nm (Supplementary Figure S6D). The free-energy minimum occurred at Rg = 1.88–1.94 nm and RMSD = 0.3–0.4 nm (Supplementary Figure S6E,F).

Collectively, these data indicate that the binding between Icaritin and EGFR remains stable throughout the molecular dynamics simulations and may possess potential biological activity.

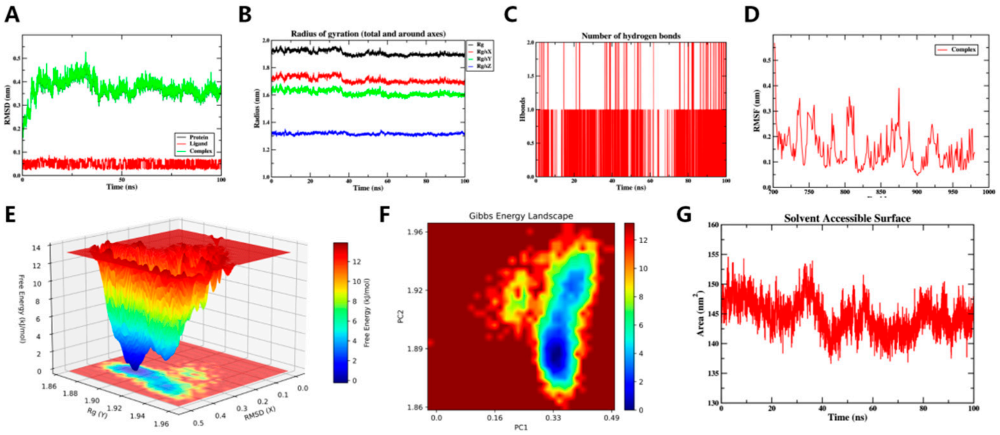

Supplementary Figure S6. The MD simulation of the Icaritin-EGFR. (A) The RMSD plot of the Icaritin-EGFR complex. (B) The Rg plot of the Icaritin-EGFR complex. (C)The number of hydrogen bonds in the Icaritin-EGFR complex. (D) The RMSF plot of the Icaritin-EGFR complex. (E) The 3D Gibbs energy landscape of the Icaritin-EGFR complex. (F) The 2D Gibbs energy landscape of the Icaritin-EGFR complex. (G) The SASA of the Icaritin-EGFR complex.

7. MD Simulation Yinyanghuo E and JUN Complexes

Equilibrium was achieved after 30 ns, with RMSD values between 0.3–0.4 nm (Supplementary Figure S7A). Rg fluctuations were minor (Supplementary Figure S7B), and SASA did not change substantially (Supplementary Figure S7G). Hydrogen-bond numbers ranged from zero to five, averaging two (Supplementary Figure S7C). Most residues exhibited low flexibility (RMSF <0.5 nm; Supplementary Figure S7D). FEL analysis identified a low-energy region at Rg = 1.72–1.78 nm and RMSD = 0.25–0.38 nm (Supplementary Figure S7E,F).

Collectively, these data indicate that the binding between Yinyanghuo E and JUN remains stable throughout the molecular dynamics simulations and may possess potential biological activity.

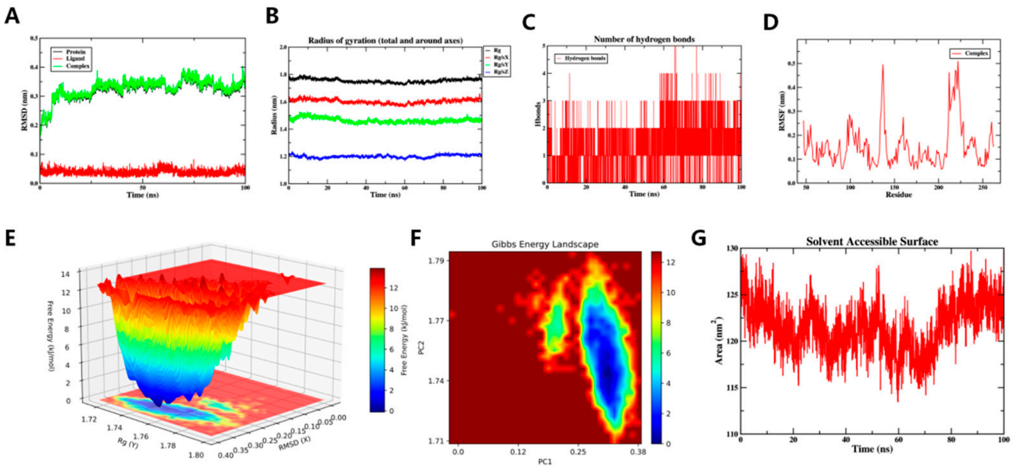

Supplementary Figure S7. The MD simulation of the Yinyanghuo E-JUN. (A) The RMSD plot of the Yinyanghuo E-JUN complex. (B) The Rg plot of the Yinyanghuo E-JUN complex. (C) The number of hydrogen bonds in the Yinyanghuo E-JUN complex. (D) The RMSF plot of the Yinyanghuo E-JUN complex. (E) The 3D Gibbs energy landscape of the Yinyanghuo E-JUN complex. (F) The 2D Gibbs energy landscape of the Yinyanghuo E-JUN complex. (G) The SASA of the Yinyanghuo E-JUN complex.

8. MD Simulation 8-Isopentenyl-kaempferol and EGFR Complexes

The RMSD stabilized within 0.3–0.4 nm after 50 ns (Supplementary Figure S8A). Rg showed limited variation (Supplementary Figure S8B), and SASA remained largely unchanged (Supplementary Figure S8G). Hydrogen-bond counts varied from zero to three, with an average of two (Supplementary Figure S8C). RMSF values were mostly below 0.5 nm (Supplementary Figure S8D). The free-energy landscape revealed a minimum at Rg = 1.88–1.94 nm and RMSD = 0.28–0.43 nm (Supplementary Figure S8E,F).

Collectively, these data indicate that the binding between 8-Isopentenyl-kaempferol and EGFR remains stable throughout the molecular dynamics simulations and may possess potential biological activity.

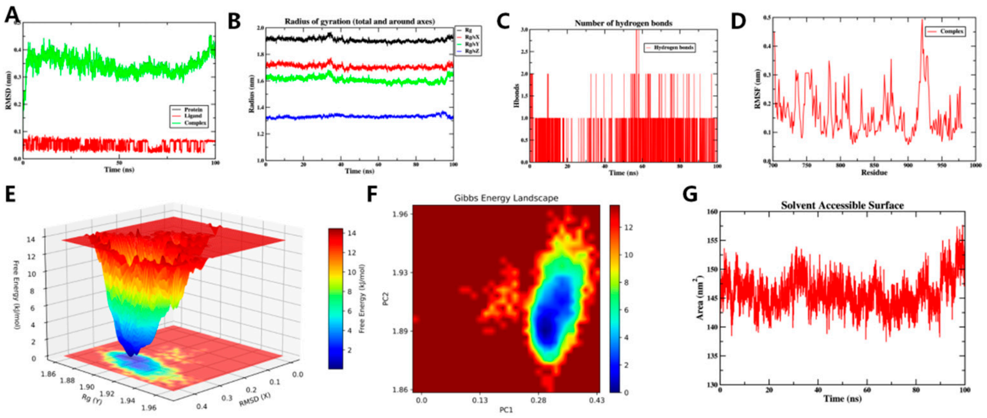

Supplementary Figure S8. The MD simulation of the 8-Isopentenyl-kaempferol-EGFR. (A) The RMSD plot of the 8-Isopentenyl-kaempferol-EGFR complex. (B) The Rg plot of the 8-Isopentenyl-kaempferol-EGFR complex. (C) The number of hydrogen bonds in the 8-Isopentenyl-kaempferol-EGFR complex. (D) The RMSF plot of the 8-Isopentenyl-kaempferol-EGFR complex. (E) The 3D Gibbs energy landscape of the 8-Isopentenyl-kaempferol-EGFR complex. (F) The

2D Gibbs energy landscape of the 8-Isopentenyl-kaempferol-EGFR. (G) The SASA of the 8-Isopentenyl-kaempferol-EGFR complex.

9. Effect of ICA adjuvant on HI-specific antibody responses

To evaluate the adjuvant efficacy of icariin in immunized mice, humoral immune responses were assessed by measuring the levels of IgG, IgG1, IgG2a, and IgG2b, as well as the IgG1/IgG2a ratio, in serum collected and isolated at 7 days post-booster immunization. To evaluate the functional relevance of the antibody responses, a challenge experiment was performed using Staphylococcus aureus USA300.

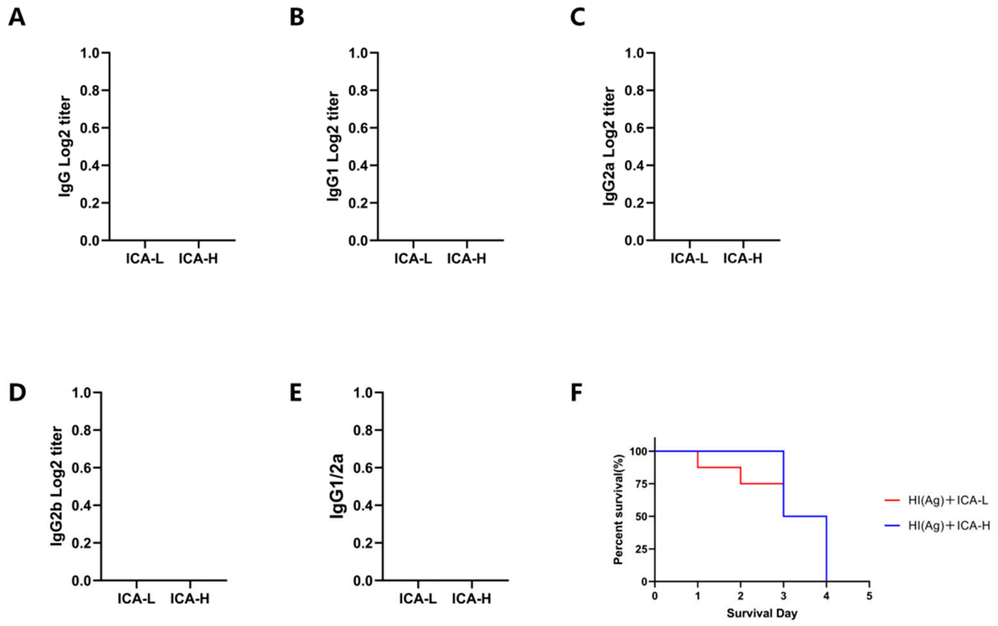

Supplementary Figure S9. Effects of ICA on HI-specific antibody levels and protection against S. aureus USA300 challenge in mice. (A-E) HI-specific IgG, IgG1, IgG2a, IgG2b, and the IgG1/IgG2a ratio in serum were measured by ELISA. (F) Survival rates of mice challenged with USA300 were monitored daily for 5 days.
